# Effect of Collagen Cross-linking on Rigid Gas Permeable Contact Lens Comfort in Keratoconus

**DOI:** 10.18502/jovr.v19i4.10891

**Published:** 2024-12-31

**Authors:** Sharafat Javaheri, Mahmood Nejabat, Asieh Ehsaei, Sahar Mohaghegh, Zahra Tajbakhsh

**Affiliations:** ^1^Poostchi Ophthalmology Research Center, Department of Ophthalmology, School of Medicine, Shiraz University of Medical Sciences, Shiraz, Iran; ^2^Department of Optometry, Mashhad University of Medical Sciences, Mashhad, Iran; ^3^Department of Optometry and visual Science, City University, London, UK; ^4^Department of Optometry, School of Rehabilitation, Shahid Beheshti University of Medical Sciences, Tehran, Iran; ^5^Department of Optometry, School of Allied Health, University of Western Australia, Perth, Australia

**Keywords:** Contact Lens Discomfort, Corneal Cross Linking, Keratoconus, Rigid Gas Permeable

## Abstract

**Purpose:**

To compare rigid gas permeable (RGP) contact lens comfort in patients with keratoconus who underwent corneal cross-linking (CXL) versus those without CXL surgery.

**Methods:**

This prospective study was carried out on 41 eyes (25 patients). Specifically, 21 eyes were assigned to the CXL group and 20 eyes to the non-CXL group. All of the patients were fitted with RGP lenses. The patients were also assessed one and three months after the initial RGP fitting. They were asked to grade themselves on a scale from 1 to 4 according to the frequency and intensity of ocular discomfort, vision fluctuation, and overall comfort with RGP lenses.

**Results:**

The mean age of participants was 24.5 ± 3.20 years. There was no significant difference in the intensity of fluctuations in vision (*P *= 0.30), frequency of discomfort (*P *= 0.29), and intensity of discomfort (*P *= 0.31) between the two groups during the one- and three-month follow-up interviews.

**Conclusion:**

Based on the current study, there is no significant disparity in self-reported discomfort with RGP contact lenses between patients with keratoconus who have undergone corneal CXL and those who have not.

##  INTRODUCTION

Keratoconus (KCN) is a bilateral ectatic disorder of the cornea, typically starting at puberty and carrying a risk of progression until the third or fourth decade of life.^[1]^ In KCN, the tensile strength of the cornea is altered without an appropriate collagen linkage. This leads to corneal destabilization, resulting in central and paracentral thinning, irregular astigmatism, myopia, and reduced visual acuity.^[2]^

Since irregular astigmatism caused by corneal ectasia cannot be sufficiently corrected by spectacles, rigid gas permeable (RGP) contact lenses are suggested as an alternative.^[3]^ Despite the availability of new RGP contact lens designs for KCN and the utilization of high oxygen permeability materials, patients with KCN do not commonly accept these types of contact lenses due to their initial discomfort.^[4]^ Besides the low acceptance rate, it is also anticipated that these patients may experience discontinuation of contact lens wear or reduced wearing time when using RGP contact lenses.

Corneal cross-linking (CXL) is a popular and effective surgical method that slows KCN progression and enhances corneal stability. These outcomes are achieved by increasing the diameter of collagen fibrils^[5]^ and strengthening their connections, in addition to reinforcing cornea's resistance against enzymatic degradation.^[6]^ CXL has been associated with significant improvements in clinical outcomes such as best-corrected visual acuity (BCVA), spherical equivalent, astigmatic correction, and corneal flattening.^[7,8]^

In this study, we aimed to evaluate whether CXL, as a therapeutic procedure in KCN, has any effect on reducing RGP lens discomfort in patients with KCN. The results will provide insight regarding the efficacy of RGP correction between CXL and non-CXL groups in clinical practice and, consequently, optimize the benefits of prescribing RGP contact lenses.

##  METHODS

### Study Design and Participants

This prospective, observational study was designed to examine RGP fitting and comfort in a group of 41 KCN eyes (25 participants, mean age ± SD = 24.5 ± 3.20 years), of which 21 underwent CXL. The number of patients who were fit monocularly in the two groups was equal (five eyes). Recruitment was conducted from 2015 to 2016 at Motahari Eye Clinic affiliated to Shiraz University of Medical Sciences, Shiraz, Iran. The study followed the Declaration of Helsinki, and the ethics approval was obtained from the local ethics committee at Mashhad University of Medical Sciences (IR.MUMS.REC.1394.589). Written informed consent was obtained from all participants at the beginning of the study.

KCN was diagnosed using slit-lamp examination, corneal tomography map (Pentacam HR, Oculus, Optikgeräte GmbH, Wetzlar, Germany), and refraction status. Individuals who did not require CXL surgery or those who had previously undergone CXL were referred to an experienced optometrist for fitting the RGP contact lenses. Participants who were scheduled to undergo CXL surgery were advised to schedule their RGP fitting at least three months after surgery. The inclusion criteria for CXL surgery were age > 19 years, corneal thickness > 400 µm, Kmax < 61 diopter (D), any change in corneal power (1 D) and refraction status, and reduction of BCVA to at least one Snellen line in the preceding year. Participants were > 18 years old and were screened for the following exclusion criteria: history of cataract, glaucoma, pterygium, meibomian gland dysfunction, blepharitis, and taking medications such as antihistamines and anti-depressants. Furthermore, participants who were not motivated to wear RGP contact lenses were also excluded from the study.

Participants were divided into the CXL and non-CXL groups. The two groups were matched for visual acuity and severity of KCN.

### Contact Lens Fitting and Interview Sessions

Uncorrected visual acuity and BCVA in high (100%) and low (20%) contrast with contact lenses were measured by Snellen chart (Itech Vision LC -13, China). Visual acuity was recorded monocularly as a decimal and then converted to logMAR for analysis. For participants with both eyes included in the study, the RGP contact lens was first fitted and assessed for the right eye, followed by the left eye. Participants provided descriptions for each eye during separate visits. Subjective refraction was performed over a trial frame based on the outcomes from an auto-refractometer (Nidek AR300, Japan) to reach the optimal visual acuity.

RGP lens was fitted by two trial sets (CFKE, CFA Wöhlk, Germany, O ${\;}_{2}$ permeability = 52 DK at 35ºC fat unit), and most participants were fitted by CFKE. The first lens was chosen based on the flat k reading (topography map) and making adjustments to the base curve to reach the acceptable three-point touch with sufficient edge clearance, appropriate lens movement, corneal coverage, and centration. All these parameters were checked by slit-lamp biomicroscope using both white and blue cobalt light. The lens with minimal residual astigmatism and visual acuity better than 0.2 logMAR was considered the optimal lens. The final lens was ordered and delivered one week later. All participants were naïve to the RGP lens and received a similar lens solution (Delta, Sauflon Company), care, contact lens insertion and removal training.

A slit-lamp biomicroscope and the Cornea and Contact Lens Research Unit (CCLRU) grading scale^[9]^ were used to grade the bulbar and palpebral hyperemia. Accordingly, eye redness was graded on a scale from 0 to 4, corresponding to no redness, very slight redness, slight redness, moderate redness, and severe redness. Tear breakup time was measured using fluorescein stripes. Two interview sessions were conducted one and three months after initiating RGP contact lens wear. During these sessions, participants were asked about the frequency of feeling discomfort while wearing the RGP contact lens and its intensity at the end of the wearing time. They were also asked and recorded about the frequency of unstable blurry vision while wearing the RGP contact lens and its intensity at the end of wearing period. Frequency scores ranged from 1 to 4 and intensity scores ranged from 1 to 5. Participants were asked to answer the following question:

“Which statement best describes your overall opinion of RGP contact lens wearing? Please select one.”

For frequency questions, the potential answers were poor (score 1), fair (score 2), good (score 3), and excellent (score 4). For intensity questions, the potential answers were never (score 1), not at all intense (score 2), slightly intense (score 3), mildly intense (score 4), and very intense (score 5). This question assessed their overall opinion about the comfort of using RGP contact lenses. The maximum number of hours participants wore contact lenses on a day was recorded, and both low- and high-contrast visual acuities were measured at each session.

### Statistical Analysis

SPSS (version 22, IBM, Armonk, New York, USA) was used for data analysis. The normality of the variables was checked using Shapiro–Wilk test. Independent samples *t*-test or Mann–Whitney U test was used to compare questionnaire scores and clinical variables in the CXL and non-CXL groups. Pearson or Spearman correlation was used to assess the associations between variables, and *P* < 0.05 was considered statistically significant.

**Figure 1:**
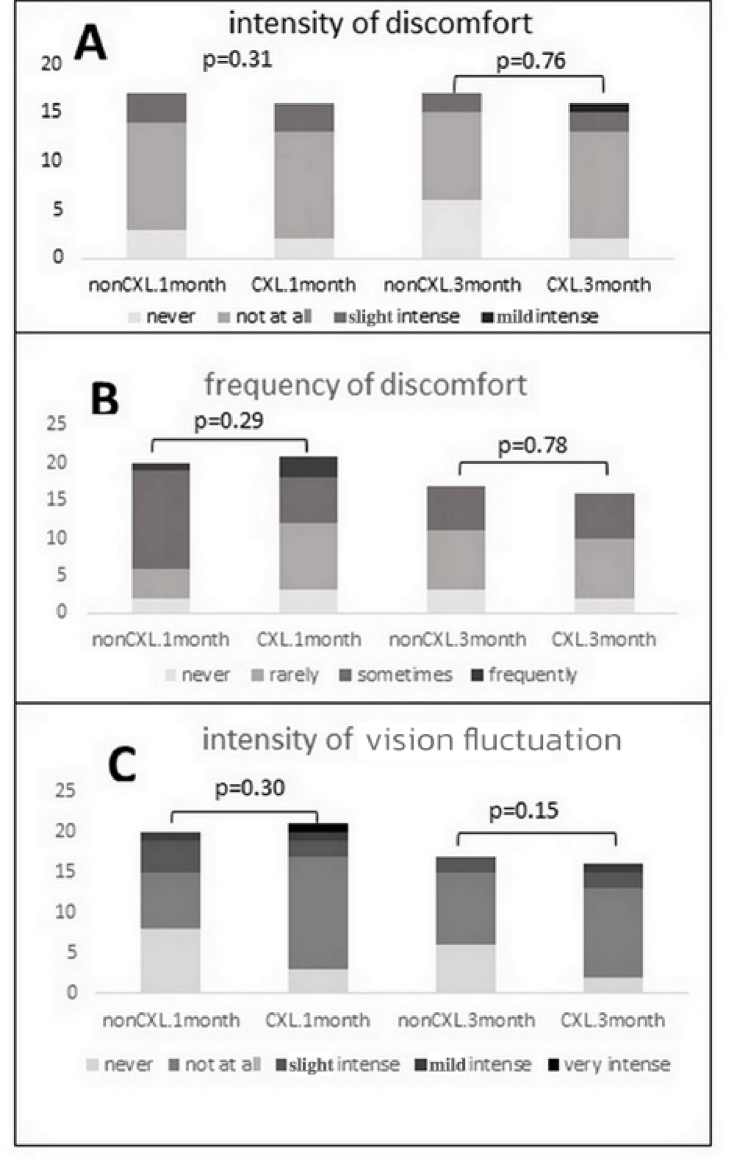
Intensity of discomfort (A), frequency of discomfort (B), and intensity of fluctuation vision (C) for the non-CXL and CXL rigid contact lens wearers at one- and three-month follow-up sessions.

**Figure 2:**
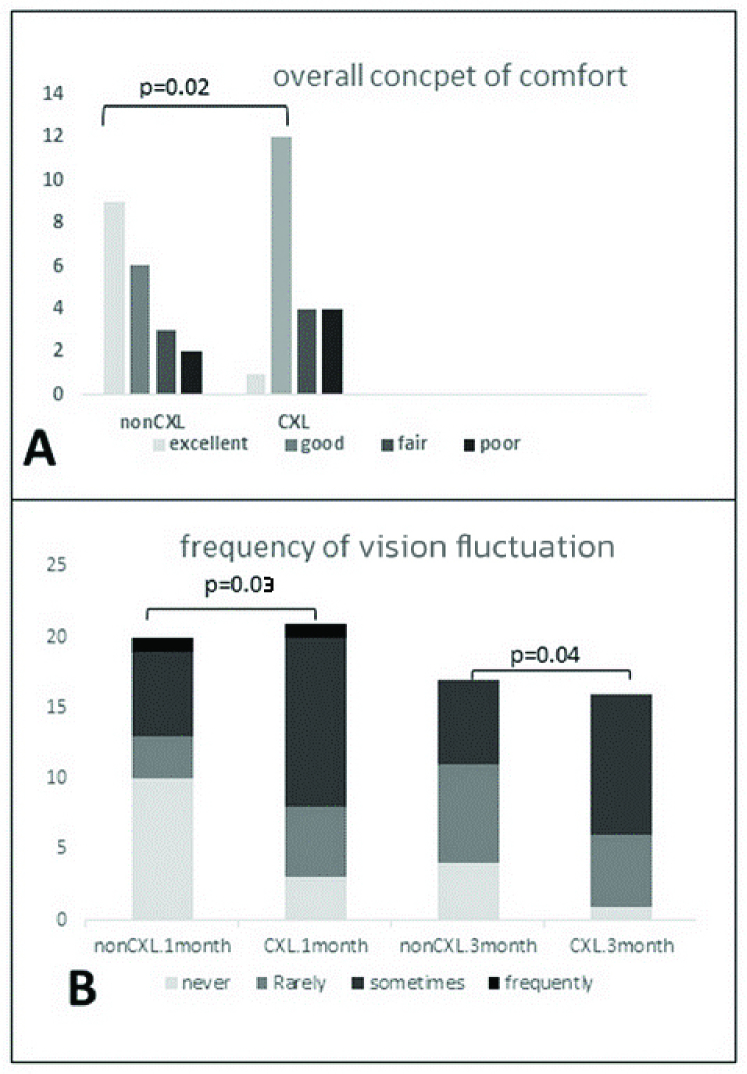
The overall concept of comfort (A) and frequency of fluctuation vision at both one- and three-month sessions (B) for non-CXL and CXL rigid contact lens wearers.

**Table 1:** Demographic characteristics and Pentacam indices for KCN and refraction in the two groups.

		**Non-CXL (** ***n*** ** = 20)**	**CXL (** ***n*** ** = 21)**	***P*** **-value**
Age (yrs)		26.7 ± 6.61	22.9 ± 5.99	0.08
Sex: Male/Female		21.1%/72.7%	78.9%/27.3%	0.002
Subjective refraction	Sphere	–3.51 ± 3.38	–3.42 ± 3.28	0.97
	Cylinder	–5.06 ± 2.34	–4.86 ± 1.65	0.66
Visual acuity (Log MAR)	BSCVA	1(0.1-1.20)	1(0.3-1.20)	0.75
	Over RGPCL Contrast 100%	0.05 (00–0.10)	0.05 (0–0.1)	0.11
	Over RGPCL Contrast 20%	0.1 (00–0.3)	0.1 (0–0.3)	0.22
Keratoconus Amsler grading	ISV	84 ± 8.0	81.14 ± 6.6	0.77
	IVA	0.73 ± 0.08	0.78 ± 0.08	0.69
	KI	1.2 ± 0.02	1.20 ± 0.02	0.89
	CKI	1.06 ± 0.01	1.06 ± 0.00	0.66
	IHA	31.1 ± 5.1	41.89 ± 6.60	0.37
	IHD	0.106 ± 0.016	0.120 ± 0.13	0.54
	RMIN	6.52 ± 0.48	6.70 ± 0.38	0.20
	TKC	2 (1–4)	2 (1–4)	0.99
Bulbar hyperemia		2 (1–4)	2 (1–4)	0.80
Palpebral hyperemia		2 (1–4)	2 (1–4)	0.70
BSCVA, best spectacle corrected visual acuity; CKI, central keratoconus index; cxl, corneal cross-linking; IHA, index of height asymmetry; IHD, index of height decentration; ISV, index of surface variance; IVA, index of vertical asymmetry; KI, keratoconus Index; logMAR, logarithm of minimum angle resolution; RMIN, minimum sagittal curvature; RRPCL, rigid gas permeable contact lens; TKC, topographic keratoconus classification; yrs, years				

**Table 2:** RGP contact lens parameters.

**Contact lens parameters**	**Non-CXL**	**CXL**
Base curve	6.9 ± 0.05	7.1 ± 0.05
Power	–4.16 ± 0.47	–3.36 ± 0.54
Diameter (9.30/9.50)	5%/95%	23.8%/76.2%
Over refraction		
Sphere	0.05 ± 0.13	0.15 ± 0.15
Cylinder	–0.88 ± 0.09	–1.01 ± 0.09
cxl, corneal cross-linking		

##  RESULTS

Twenty-five participants completed the study. While 9 participants were included unilaterally, 16 others were included bilaterally. The Cronbach's alpha for the maximum keratometry was 0.6 (*P *= 0.04). Twenty-one eyes were assigned to the CXL group and 20 eyes into the non-CXL group. Table 1 summarizes the demographic characteristics and Pentacam indices for KCN and refraction in the two groups. The mean K ${\;}_{max}$ values were 58.20 ± 2.40 D and 57.90 ± 2.70 D in the CXL and non-CXL groups, respectively, which was not significantly different between the two groups (*P *= 0.62). The KCN classification indices, measured by Pentacam, were not significantly different between the two groups (all *P* < 0.05). The non-CXL group had a higher proportion of female participants (*P *= 0.002), and there was no significant difference in age between the two groups (*P *= 0.08).

### Interview Session Outcome

According to participants' responses, there were no differences between the CXL and non-CXL groups in terms of factors such as frequency of discomfort during wearing time (*P *= 0.29), intensity of discomfort at the end of wearing time (*P *= 0.31), and intensity of vision fluctuation at the end of wearing time (*P *= 0.30) in either the one- or three-month follow-up session [Figure 1]. The frequency of vision fluctuation was lower in the non-CXL group than in CXL group at both one- and three-month follow-up visits (*P *= 0.03 and *P *= 0.04, respectively) [Figure 2]. The question regarding the overall concept of comfort in RGP lenses also revealed a significant difference between the two groups (*P *= 0.02), with the non-CXL group reporting more comfort with the RGP lenses [Figure 2].

The RGP contact lens parameters, including base curve, power, and over-refraction, were not significantly different between the two groups [Table 2]. The mean wearing time was 8.80 ± 2.8 hours in the non-CXL and 7.71 ± 3.06 hours in the CXL group, indicating no significant difference between the two groups (*P *= 0.24). In the non-CXL group, three participants discontinued wearing RGP contact lenses after one month, whereas five participants in the CXL group discontinued wearing RGP contact lenses during the same period.

As expected, the visual acuity of participants was significantly better with RGP contact lenses than with spectacle correction. Furthermore, low-contrast visual acuity was slightly lower than high-contrast visual acuity. There was no significant difference between visual acuity with RGP contact lenses at baseline and three months later (*P *= 0.08).

According to the CCLRU grading scale, ocular surface indices, including bulbar and palpebral hyperemia, were predominantly mild in both non-CXL and CXL groups, with no significant difference between the two groups (*P *= 0.80 and *P *= 0.70, respectively). Finally, it is worth mentioning that the mean tear breakup time was slightly higher in the CXL group.

##  DISCUSSION

In this study, the frequency and intensity of discomfort and the intensity of vision fluctuation were similar in the two groups. However, participants in the CXL group experienced vision fluctuation more frequently and exhibited a lower overall comfort with RGP lenses.

There is limited information about the relationship between CXL and the comfort of RGP contact lenses. Ünlü et al indicated that tolerance of RGP contact lenses tends to improve following CXL surgery, possibly due to reduced corneal sensitivity and corneal curvature.^[10]^ The methodology employed in their study differed from the present study as they specifically examined tolerance for RGP contact lens in individuals who initially experienced intolerance to RGP contact lenses both before and one month after undergoing CXL. On the other hand, another study showed that CXL surgery did not have any effect on tolerance toward scleral contact lenses.^[11]^

In the current study, the RGP contact lens fitting was based on the three-point touch philosophy, and contact lens parameters including base curve and diameter and power were almost the same across the two groups. We did not observe any changes in RGP fit after CXL. However, previous reports have highlighted certain changes in the cornea-contact lens-fitting relationship due to the changes in the shape and position of the cone apex after CXL.^[12]^ These changes have been observed despite using the same contact lens parameters.^[12]^ Moreover, Singh et al noted a tendency toward a flatter lens fit in all patients who had undergone CXL. Specifically, the authors observed an increase in the percentage of optimal and acceptable fit, which they attributed to the corneal compactness post-CXL.^[13]^

In our study, the mean wearing time was 7.71 hours/day in the CXL group and 8.80 hours/day in the non-CXL group. This is consistent with other studies reporting wearing times ranging from 10.36 to 8 hours/day.^[12,13]^ Another study demonstrated that wearing time before CXL was 6.4 hours/day and increased to 13.2 hours/day by six months post-CXL.^[10]^

Better visual acuity achieved through RGP contact lenses can motivate participants to wear them more frequently and for longer durations. In our study, there were no significant differences in visual acuity with RGP contact lenses between the CXL and non-CXL groups. Similarly, another study has reported comparable visual acuity with contact lenses before and after CXL.^[12]^

In the current study, the dropout rate for contact lens wear was 23.8% in the CXL and 15% in the non-CXL groups. Five participants in the CXL group discontinued wearing contact lenses. Reasons for discontinuation varied: one participant preferred wearing spectacles due to similar visual acuity with both spectacle and RGP; another had severe hyperemia and a history of vernal keratoconjunctivitis (VKC); one experienced constant vision fluctuation; and two participants lacked motivation and sought long-term treatments such as intracorneal ring segment implantation.

The optimal timing for fitting the RGP contact lens after CXL is still unknown. Different studies have proposed different times ranging from one week and one month to six months. ${\;}^{\left[\text{13,15,20}\right]}$ Studies have shown that corneal sensitivity temporarily decreases after CXL and typically returns to the preoperative level within 6 to 12 months.^[16,17]^ This finding suggests that RGP contact lens fitting after six months post-CXL could offer better outcomes. In the current study, contact lens fitting was performed at least three months after CXL, and comfort assessments were performed after one and three months of initiating contact lens wear. All participants in our study were new to wearing RGP contact lenses, which could potentially impact the comfort rate. However, we conducted the interview session with participants after one month, which should have allowed sufficient time for adaptation. In one study, the average adaptation time of 10 days was reported for RGP contact lenses.^[18]^ Further studies are needed to confirm the ideal timing for RGP contact lens fitting after CXL.

In the present study, the severity of KCN was assessed using the Amsler-Krumeich classification system. Findings from the CLEK study^[19]^ indicated that the severity of KCN, as determined by steep keratometry, did not have a significant impact on the level of patient's comfort with rigid lenses. Another study highlighted that quality of life is primarily influenced by binocular visual acuity rather than keratometric values. ${\;}^{\text{[20]}}$ Besides, in the present study, the discomfort rate was not measured based on the severity of KCN. Therefore, a study with a larger sample size is required to evaluate the rate of discomfort associated with different levels of KCN severity. The two groups were well-matched in terms of clinical performance, including visual acuity and KCN severity; however, the two groups were not matched in terms of gender distribution. Meanwhile, the inclusion of a non-CXL control group was a strength of the present study.

In summary, there is no important difference in self-reported discomfort from RGP contact lenses between patients with KCN who have undergone CXL and those who have not. These findings highlight the need for more research to confirm the impact of CXL on the acceptance of RGP contact lenses.

##  Financial Support and Sponsorship

None.

##  Conflicts of Interest

None.
